# Added Value of Computed Tomography Virtual Intravascular Endoscopy in the Evaluation of Coronary Arteries with Stents or Plaques

**DOI:** 10.3390/diagnostics12020390

**Published:** 2022-02-03

**Authors:** Patricia Wanping Wu, Pei-Kwei Tsay, Zhonghua Sun, Syu-Jyun Peng, Chia-Yen Lee, Ming-Yi Hsu, Yu-Shien Ko, I-Chang Hsieh, Ming-Shien Wen, Yung-Liang Wan

**Affiliations:** 1 Department of Medical Imaging and Intervention, Linkou Chang Gung Memorial Hospital, College of Medicine, Chang Gung University, Taoyuan City 333423, Taiwan; pwwu@cgmh.org.tw (P.W.W.); m7259@cgmh.org.tw (M.-Y.H.); 2 Department of Public Health and Center of Biostatistics, College of Medicine, Chang Gung University, Taoyuan City 333323, Taiwan; tsay@mail.cgu.edu.tw; 3 Discipline of Medical Radiation Science, Curtin Medical School, Curtin University, Bentley, WA 6102, Australia; Z.Sun@exchange.curtin.edu.au; 4 Professional Master Program in Artificial Intelligence in Medicine, College of Medicine, Taipei Medical University, Taipei City 110301, Taiwan; sjpeng2019@tmu.edu.tw; 5 Department of Electrical Engineering, National United University, Miaoli 360302, Taiwan; olive.ntu@gmail.com; 6 Department of Cardiology, Linkou Chang Gung Memorial Hospital, College of Medicine, Chang Gung University, Taoyuan City 333423, Taiwan; c12037@cgmh.org.tw (Y.-S.K.); hsiehic@ms28.hinet.net (I.-C.H.); wenms123@cgmh.org.tw (M.-S.W.)

**Keywords:** coronary artery disease (CAD), coronary computed tomography angiography (CCTA), virtual intravascular endoscopy (VIE), coronary stent, coronary calcification

## Abstract

Coronary computed tomography angiography (CCTA) is a widely used imaging modality for diagnosing coronary artery disease (CAD) but is limited by a high false positive rate when evaluating coronary arteries with stents and heavy calcifications. Virtual intravascular endoscopy (VIE) images generated from CCTA can be used to qualitatively assess the vascular lumen and might be helpful for overcoming this challenge. In this study, one hundred subjects with coronary stents underwent both CCTA and invasive coronary angiography (ICA). A total of 902 vessel segments were analyzed using CCTA and VIE. The vessel segments were first analyzed on CCTA alone. Then, using VIE, the segments were classified qualitatively as either negative or positive for in-stent restenosis (ISR) or CAD. These results were compared, using ICA as the reference, to determine the added diagnostic value of VIE. Of the 902 analyzed vessel segments, CCTA/VIE had sensitivity, specificity, accuracy, positive predictive value, and negative predictive value (shown in %) of 93.9/90.2, 96.2/98.2, 96.0/97.7, 70.0/83.1, and 99.4/99.0, respectively, in diagnosing ISR or CAD, with significantly improved specificity (*p* = 0.025), accuracy (*p* = 0.046), and positive predictive value (*p* = 0.047). VIE can be a helpful addition to CCTA when evaluating coronary arteries.

## 1. Introduction

With rapid technological advancements, coronary computed tomography angiography (CCTA) has become an increasingly useful tool in the diagnosis of coronary artery disease (CAD) [1,2,3]. Its improved spatial and temporal resolution is reflected in the high diagnostic accuracy achieved with multi-detector computed tomography (CT) scanners (64-slice and higher). CCTA is now regarded as a reliable modality for diagnosing CAD in patients with low to intermediate risk of CAD; its high specificity and negative predictive value make it a highly useful screening tool, reducing unnecessary invasive procedures [4,5,6,7,8]. However, one of the main limitations of CCTA is that its diagnostic value, especially its specificity and positive predictive value, is relatively low, and is significantly decreased in patients with coronary stents or heavy calcifications (Agatston score > 400) due to blooming artifacts, which result in high false positive rates [9,10,11,12,13].

Meta-analyses of CCTA performed on 64-slice multi-detector CT scanners showed a sensitivity of 86–91% and specificity of 91–93% in diagnosing in-stent restenosis (ISR), while the positive predictive value remained low at 68% [14,15,16]. It has been reported that 320-row CCTA had a specificity of 83–96% and a positive predictive value of 46–74% in diagnosing ISR [17,18]. On patient-based analysis, the specificity of CCTA decreases as the calcium score (CS) increases; the specificity is 92.9% for patients with zero CS, 83.3% when CS is between 11 and 100, and 60% when CS is between 401 and 1000 [9], indicating the negative impact of coronary artery calcifications on the diagnostic performance of CCTA. This presents a challenge for CCTA because the low specificity could lead to invasive procedures that could have been avoided in these patients.

Virtual intravascular endoscopy (VIE), a CCTA-generated 3D visualization tool, is another approach that provides unique intraluminal views of the blood vessels, including endovascular stents or stent grafts [19,20,21,22,23,24,25,26,27,28], thus having potential to overcome the limitations of CCTA. To our knowledge, there are very few reports investigating the application of virtual intravascular endoscopy (VIE) in assessing coronary arteries [25,26,27,28]. Only one study has compared the diagnostic performance of VIE with that of CCTA, via an investigation of 61 patients with calcified coronary plaques [26]. Another article has described using VIE to assess coronary arteries with stents, but the case number is limited to only 13 patients [27].

The purpose of this study is to investigate the role of VIE in delineating coronary intraluminal changes in the presence of coronary stents and plaques. By performing both CCTA and invasive coronary angiography (ICA) for each patient within 4 days, the added diagnostic value of VIE was compared with that of CCTA alone, using ICA as the gold standard.

## 2. Materials and Methods

### 2.1. Study Population

The Institutional Review Board approved this project. For a prior prospective study assessing the diagnostic value of CCTA using a 320-detector CT scanner, 100 patients with previously placed coronary stents were recruited from a single tertiary medical hospital to undergo both CCTA and ICA within a 4-day time interval. Informed consent forms were obtained from all subjects. The exclusion criteria comprised the following: previous history of cardiac bypass surgery; adverse reactions to iodinated contrast media; renal insufficiency with glomerular filtration rates of less than 45 mL/min/1.73 m^2^; difficulty in cooperating with the aims of the study; hemodynamic instability; and severe tachycardia or arrhythmias. The subjects’ CCTA and ICA images were then used retrospectively for this study.

### 2.2. CT Techniques for Coronary Calcium Scoring and CCTA

All patients underwent both non-enhanced CT and CCTA on a 320-detector CT scanner (Aquilion ONE, Toshiba Medical Systems, Otawara, Japan) with 320 detector rows, each 0.5 mm wide, and a gantry rotation time of 0.35 s with z-axial coverage of up to 160 mm. Calcium scores were measured on non-enhanced CT images, assessed and recorded on a separate workstation (Vitrea FX, Vital Images, Inc., Minnetonka, MIN, USA). Slice thickness was set at 3 mm for Agatston scoring and 1 mm for CCTA, with the coverage area extending from the pulmonary trunk to the diaphragm. The CT images were obtained based on a standard protocol [18,29,30].

Tube voltage (kV) and current (mA) were selected according to the patients’ BMI. Kernel settings FC03 and FC05 were used. For vascular enhancement, a bolus of contrast medium (Iohexol; Omnipaque 350, GE Healthcare, Cork, Ireland) was administered intravenously, with a volume of 60 to 70 mL at a rate of 5 mL/s through the antecubital vein using a power injector (Mallinckrodt LF OptiVantage DH V8402, Cincinnati, OH, USA), followed by 40 mL of saline chase at a rate of 5 mL/s. CT scanning was started manually when contrast medium reached peak attenuation in the left ventricle.

Before the scan, a β-blocker Inderal (Propranolol Hydrochloride 10 mg/tab, AstraZeneca UK Ltd., Chesire, UK) was administered orally to keep the heart rate below 65 bpm for the one-beat scan. A short-acting cardio-selective β-blocker, Esmolol (Esmolol solution, Esmolol HCL 10 mg/mL, JenYa Biotech Inc. Ltd., Hsing Chu, Taiwan) was administered intravenously if there were any contraindications to Inderal, such as asthma, atrioventricular conduction block, and Raynaud syndrome. For dilatation and optimal visualization of the coronary arteries, 0.6 mg of Nitrostat (Nitroglycerin 0.6 mg/tab, Pfizer Pharmaceuticals LLC, Puerto Rico, USA) was administered sublingually 3–5 min before the scan.

### 2.3. Invasive Coronary Angiography

**Figure 1:**
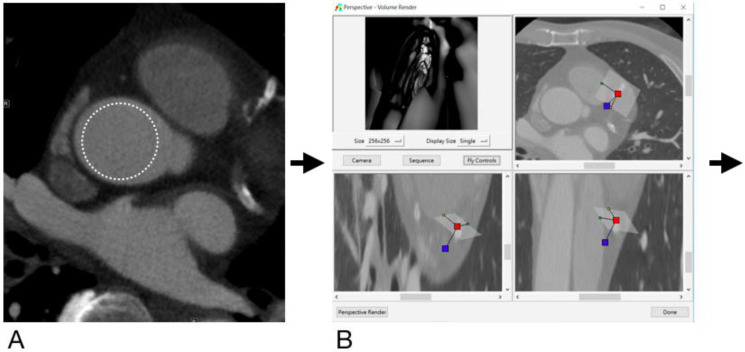

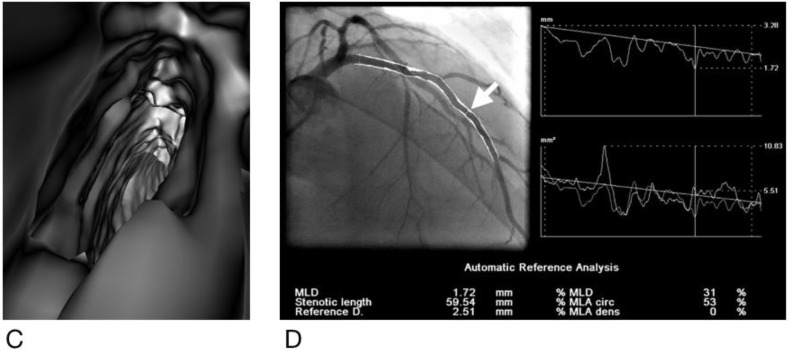
Flowchart showing image post-processing steps from original DICOM images to generating VIE images. The CT attenuation is measured in the ascending aorta on original DICOM images (**A**) to set the approximate threshold for generation of VIE (**B**), revealing intimal hyperplasia in a stent placed in the proximal- to middle- left anterior descending artery (**C**). This is then compared with the results of invasive coronary angiography (**D**), which also shows intimal hyperplasia with 31% stenosis (arrow).

All ICA was performed within 4 days after CCTA. All images were recorded at 15 frames/s by a monoplane X-ray angiogram (AXIOM-Artis, Siemens, Forchheim, Germany) at a resolution of 512 × 512 pixels. The ICA studies were performed and analyzed by two experienced cardiologists blinded to the CCTA data. Minimal lumen diameters were quantitatively measured in projections showing the most severe narrowing, using a quantitative analysis software package (Scientific QCA Analysis, Siemens, Forchheim, Germany), as shown in Figure 1. A stenosis of more than 50% was considered to be ISR if the stenosis was located within 0.5 cm of a stented vessel segment [31], or CAD if the stenosis was found in a native vessel.

### 2.4. Generation of Virtual Intravascular Endoscopy Images

CT volume data converted from original DICOM (digital imaging and communications in medicine) images were transferred to a separate workstation equipped with the software Analyze V 11.0 (AnalyzeDirect, Inc., Overland Park, KS, USA) for image post-processing and generation of 3D VIE images. Post-processing of CT data was performed with a CT number thresholding technique [27,28]. Generation of VIE images of coronary plaques and coronary lumen depends on the selection of an appropriate CT threshold, which was determined by measuring the CT attenuation in the ascending aorta according to previous reports [23,24,25], as illustrated in Figure 1. An upper CT threshold of 150 to 200 HU was applied to remove the contrast-enhanced blood from the coronary artery for optimal demonstration of intraluminal views of coronary walls. 

### 2.5. Analysis and Interpretation of CCTA and VIE Images

The minimal luminal diameters were quantitatively measured on curved planar reformatted CCTA images, in views showing the greatest degree of stenosis in the left anterior descending (LAD), left circumflex (LCX), and right coronary (RCA) arteries. All vessels greater than 1.5 mm in diameter were evaluated [8]. A stenosis of more than 50% was considered to be ISR if the stenosis was located within 0.5 cm of a stented vessel segment, or CAD if the stenosis was found in a native vessel. The presence of calcified, mixed, and non-calcified plaques was recorded.

VIE interpretation was performed jointly by 2 observers (with 6 and 15 years of experience in cardiac CT imaging, respectively) who were blinded to the results of ICA, but not CCTA, because CCTA images are required to generate the VIE images and locate the vessel segments. Thirty patients’ CCTA and ICA images were used to train both observers to construct and interpret VIE images, as well as to determine the inter- and intra-observer variability, before proceeding with the main study. During this training session, the observers learned to choose appropriate CT thresholds in order to generate VIE images of optimal quality, so that the degree of stenosis in each vessel matched their appearance on CCTA images under most circumstances (especially in vessels without extensive artifacts). The LAD, LCX, and RCA were each divided into proximal, middle, and distal segments for VIE analysis. The left main coronary artery, posterior descending artery, and other branches (such as diagonal and obtuse marginal branches) were included only if they had stents or suspected CAD on CCTA. Vessel segments were excluded if they could not be evaluated on VIE due to hypoplasia or a location distal to totally occluded segments.

The VIE images were interpreted qualitatively, categorizing each vessel segment as either negative or positive for CAD or ISR; vessel segments were considered positive if the lumen appeared to be more than 50% obstructed, either by filling defects or circumferential narrowing. If the degree of stenosis was visually equivocal (more or less 50%), the diagnosis of VIE generally followed that of CCTA. The diagnostic value of CCTA alone is compared with that of CCTA-based VIE (which, for simplicity, will be referred to as VIE in the rest of this article), using ICA results as the gold standard.

### 2.6. Statistical Analysis

All quantitative variables were expressed as mean ± standard deviation and compared by Student’s *t*-test. Categorical variables were expressed as numbers and percentages and analyzed by Chi-square or Fisher’s Exact test. Sensitivity, specificity, accuracy, positive predictive value, and negative predictive value for original CCTA and VIE images in detecting stenosis in coronary arteries with stents or calcifications were calculated from χ^2^ tests of contingency. Kappa values were used to assess inter- and intra-observer variability, and to estimate the degree of agreement between CCTA, VIE and ICA in diagnosing coronary stenosis. A *p* value of less than 0.05 was considered significant for all statistical evaluations.

## 3. Results

Between November 2010 and July 2012, one hundred patients with coronary stents agreed to undergo both CCTA and ICA examinations. All subjects except one underwent both CCTA and ICA within a one-day interval; one subject underwent ICA four days after CCTA. Of these 100 subjects (mean age 55.3 years, ranging from 35 to 74 years), 91 were males. All patients had at least one coronary stent placed in the main coronary arteries. The average total Agatson’s calcium score was 249.5 (range 0 to 2928).

**Figure 2:**
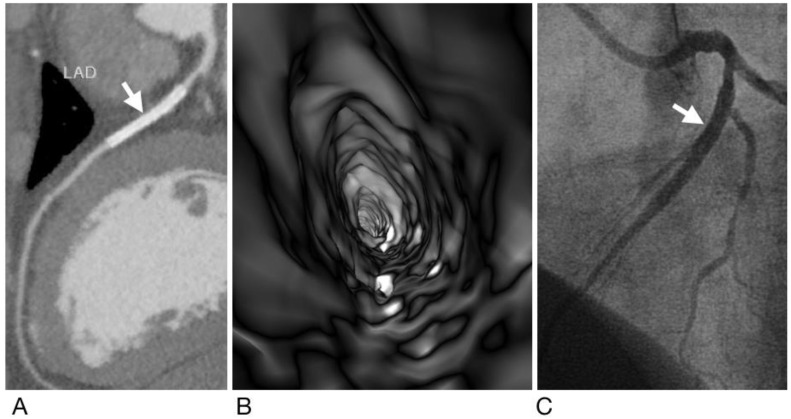
A case of true negative results for both CCTA and VIE. Subject is a 45-year-old woman with hypertension and type 2 diabetes mellitus and has a metallic stent (Xience V, 2.5 mm × 38 mm) (arrow) placed in the left anterior descending artery (LAD). Both CCTA (**A**) and VIE (**B**) showed patency of the stent without ISR, which was confirmed by ICA (arrow) (**C**).

**Figure 3:**
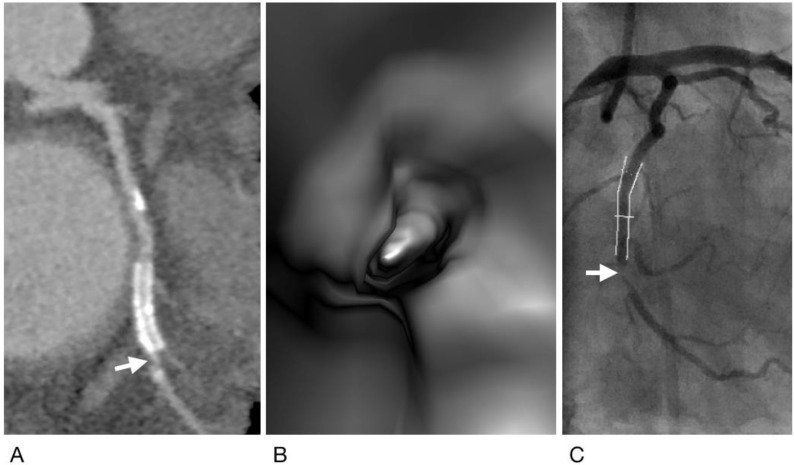
A case of true positive result for both CCTA and VIE. Subject is a 57-year-old man with hypertension and obesity (body mass index = 30.7 kg/m^2^) and with a total calcium score of 1125. A metallic stent was placed in the left circumflex artery. Both CCTA (**A**) and VIE (**B**) showed ISR at the distal edge of the stent (arrow). This was confirmed by ICA (**C**), showing 88% stenosis (arrow).

**Figure 4:**
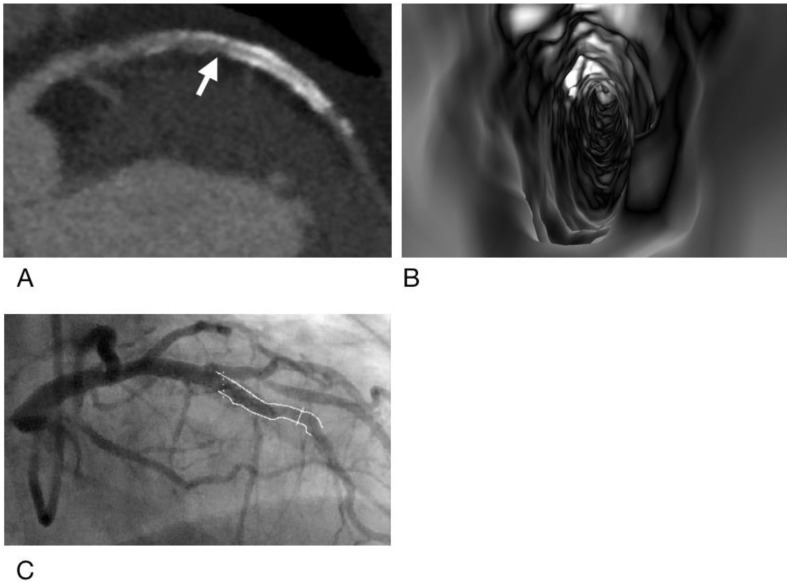
A case of false positive result for CCTA but true negative result for VIE. Subject is a 51-year-old man with obesity (body mass index = 32.8 kg/m^2^) and a total calcium score of 815. A metallic stent (Xience V, 2.5 mm × 23 mm) was placed in the left anterior descending artery. CCTA (**A**) showed severe calcifications with poor opacification at the proximal end of the stent (arrow) suggestive of ISR, but VIE (**B**) found only intimal hyperplasia. ICA (**C**) showed only intimal hyperplasia with 12% stenosis in this stent.

All vessel segments with either stents or suspected CAD seen on CCTA were analyzed using VIE. In total, 902 vessel segments, including 193 stented vessel segments and 231 plaque segments, including 115 calcified plaque segments, 85 mixed plaque segments, and 31 non-calcified plaque segments, were analyzed. Some examples of CCTA, VIE, and ICA images in corresponding vessel segments are shown in Figure 2, Figure 3 and Figure 4.

### 3.1. Inter- and Intra-Observer Variability

The intra-observer agreement was weak for both observers (kappa = 0.584 and 0.414, respectively) when comparing the results from the first 30 training cases with the second-round interpretation. Inter-observer agreement was moderate after the training round was completed (kappa = 0.726).

### 3.2. Diagnostic Performance of CCTA and VIE

Overall, out of a total of 902 analyzed vessel segments (Table 1), 82 (9.1%) segments were proven to have significant ISR or CAD by ICA. CCTA/VIE had sensitivity, specificity, accuracy, positive predictive value, and negative predictive value (shown in %) of 93.9/90.2, 96.2/98.2, 96.0/97.7, 70.0/83.1, and 99.4/99.0, respectively, in diagnosing ISR or CAD, with significantly improved specificity (*p* = 0.025), accuracy (*p* = 0.046), and positive predictive value (*p* = 0.047). The kappa value for CCTA agreement with ICA in diagnosing ISR or CAD showed an overall improvement from 0.779 to 0.851 following the addition of VIE interpretation, although this change did not reach statistical significance (*p* = 0.112). 

Of the 193 stented vessel segments, 22 (11.4%) were proven to have ISR by ICA. CCTA had a sensitivity, specificity, accuracy, positive predictive value, and negative predictive value (shown in %) of 95.7, 93.5, 93.8, 66.7, and 99.4, respectively, in diagnosing ISR. With VIE, the corresponding values (shown in %) were 87.0, 97.6, 96.9, 83.3, and 98.2, respectively, for diagnosing ISR (Table 2). The specificity, accuracy, and positive predictive value seemed to be slightly improved, although these changes did not reach statistical significance.

Of the 115 vessel segments with calcified plaques, four (3.5%) were proven to have CAD by ICA. For these segments, CCTA/VIE had sensitivity, specificity, accuracy, positive predictive value, and negative predictive value (shown in %) of 75.0/50.0, 88.3/95.5, 87.8/93.9, 18.8/28.6, and 99.0/98.1, respectively, in diagnosing CAD (Table 3). The specificity and accuracy seemed to be partially improved, although again this change did not reach statistical significance. 

Of the 85 vessel segments with mixed plaques, 34 (40.0%) were proven to have CAD by ICA. For these segments, CCTA/VIE had sensitivity, specificity, accuracy, positive predictive value, and negative predictive value (shown in %) of 91.2/91.2, 88.2/92.2, 89.4/91.8, 83.8/88.6, and 93.8/94.0, respectively, in diagnosing CAD (Table 4). There was no significant difference between the diagnostic values of CCTA and VIE in detecting CAD in vessel segments with mixed plaques. 

Of the 31 vessel segments with non-calcified plaques, 21 (67.7%) were proven to have CAD by ICA. For these segments, CCTA/VIE had sensitivity, specificity, accuracy, positive predictive value, and negative predictive value (shown in %) of 100.0/100.0, 70.0/80.0, 94.3/93.6, 87.5/91.3, and 100.0/100.0, respectively, in diagnosing CAD, without any statistically significant difference (Table 5).

Overall, for the combined 424 vessel segments with stents and plaques, CCTA/VIE had sensitivity, specificity, accuracy, positive predictive value, and negative predictive value (shown in %) of 93.9/90.2, 90.4/95.6, 91.0/94.6, 70.0/83.1, and 98.4/97.6, respectively, in diagnosing CAD (Table 6). The specificity and positive predictive value were significantly improved (*p* = 0.011 and *p* = 0.047, respectively).

### 3.3. Effect of Stents on the Diagnostic Performance of CCTA and VIE

Stent details were available for 190 out of the 193 stented vessel segments, including 144 drug-eluting stents and 46 bare-metal stents. The stents ranged from 1.3 mm to 5 mm in diameter. Smaller stent diameters were significantly associated with ISR in CCTA (*p* = 0.001), VIE (*p* < 0.001), and ICA (*p* < 0.001). Smaller stent diameters also resulted in significantly decreased diagnostic accuracy for both CCTA (*p* = 0.004) and VIE (*p* < 0.001), although there was no significant difference in this effect between CCTA and VIE (*p* = 0.547). Stent types (bare-metal stents or drug-eluting stents) had no significant effect on ICA results (*p* = 0.822).

## 4. Discussion

One of the main limitations of CCTA is the decrease in its diagnostic accuracy in the presence of high-absorption materials such as calcifications and stents. The evaluation of stented or calcified vessels presents challenges for CCTA due to the presence of blooming artifacts, which lead to relatively low specificity and positive predictive value [9,10,11,12,13]. VIE generated from CCTA data provides unique intraluminal views that allow qualitative assessment of the arterial lumen. Several other studies have used VIE to evaluate the aorta [19,20,21,22,23,24], but only three studies have used VIE to assess coronary arteries [25,26,27]. Only one study has evaluated the diagnostic value of VIE in assessing coronary stenosis, via an investigation of 61 patients with calcified coronary plaques, and showed a significant improvement in specificity and positive predictive value when compared with CCTA [26]. To our knowledge, the present study is the largest series to have assessed the added value of VIE in coronary artery evaluation—including stented, calcified plaques, mixed plaques and non-calcified plaques—compared with the diagnostic value of CCTA alone, using ICA as the reference method. 

The results of our study showed that the use of VIE was able to significantly improve the specificity, accuracy, and positive predictive value of CCTA when evaluating coronary arteries, without sacrificing the already high sensitivity and negative predictive value of CCTA. This appears to be especially promising in vessels with stents and plaques, which were assessed with significantly improved specificity (*p* = 0.011). Of the 902 vessel segments analyzed in this study, CCTA had 33 false positive results, among which VIE was able to correctly identify 25 (75.8%) as true negative results (Figure 4). These 25 segments comprised 11 stents, eight calcified plaque segments, four soft plaque segments and two mixed plaque segments. The remaining eight segments, which also received false positive results by using VIE, comprised five calcified and three mixed plaque segments.

The increased specificity, accuracy, and positive predictive value of VIE may be attributed to the CT number thresholding technique, which is able to remove both enhanced blood and abnormal blooming artifacts from the VIE images. However, during VIE image interpretation, the threshold often requires manual adjustment to avoid under-deletion of image data, which would result in increased false positive findings, and over-deletion, which can result in the appearance of a “perforated” vessel wall. As mentioned in our Methods section, the threshold for VIE image construction was set between 150 and 200HU. When generating VIE images, we usually begin with a threshold of 200 HU, then gradually adjust to lower thresholds if a vessel that is clearly patent on CCTA appears narrowed on VIE. This is based on the assumption that CCTA has a high negative predictive value, and thus we should adjust the threshold so that the VIE images match the CCTA images in negative vessel segments. On the other hand, when the threshold is too low, the vessel walls get deleted and will appear perforated (this tends to occur in the LCX, as it is very close to the left atrium); in such instances, the threshold needs to be set higher. With some practice, observers can become adequately adept at choosing the appropriate threshold and constructing quality VIE images for interpretation. 

**Figure 5:**
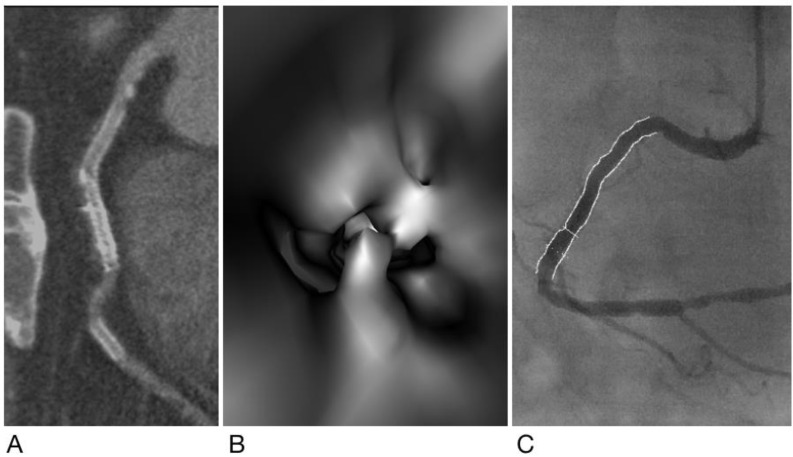
A case of true negative result for CCTA but false positive result for VIE. Subject is a 72-year-old man with hypertension and with 3 metallic stents placed in the right coronary artery. CCTA (**A**) showed intimal hyperplasia in all three stents without ISR, but VIE (**B**) found ISR in the middle stent. ICA (**C**) showed only intimal hyperplasia with 34% stenosis in the middle stent.

In our study, there were seven vessel segments (four stented vessel segments and three native vessel segments with either mixed or non-calcified plaques) that appeared to be ISR or CAD on VIE images, but were diagnosed as only intimal hyperplasia (IH) or mild stenosis on both CCTA and ICA (an example is shown in Figure 5); these segments were either located at the distal edges of stents or in distal or small caliber vessels. Tortuous vessels, long segmental stenoses, and small caliber vessels are all pitfalls during VIE image interpretation. Thus, it may be deduced that VIE may provide more value when evaluating larger and more proximal vessel segments, but becomes less useful when evaluating smaller and more distal vessel segments. It should be emphasized that because VIE images are created using CCTA images, there is no need to interpret VIE images alone, either in a clinical setting or in this study. In cases of difficult vessel segments, CCTA images should be reviewed again to obtain a consensus. 

Another limitation of this technique is that the use of appropriate software to generate VIE images has a learning curve, requiring several steps and some decision-making. Thin slice images (0.5 mm axial) are required for optimal VIE image generation. Assessing all of the coronary artery segments in a patient without referring to CCTA images is time-consuming and may require well over an hour per patient. Since CCTA already has a high negative predictive value (97.8% in a previously published meta-analysis [32]), a more practical approach is to evaluate the vessels on CCTA first, and then recheck the suspected lesions using VIE for confirmation, especially in segments with stents and heavy calcifications. In our experience, the time spent on assessing each patient using CCTA and VIE together depends largely on the number of suspected CAD/ISR lesions, image quality, and familiarity with the software. On average, each vessel takes about 5 min to evaluate on CCTA and VIE together.

In addition to the limitations of VIE, this study has some limitations of its own. This is a single-center study, with only 100 subjects and 902 evaluated vessel segments—in particular, the number of positive vessel segments with calcified plaques is small, only four. Based on our results, a larger scale study might be expected to produce more statistically significant results. Second, the learning curve of the VIE software may have affected the interpreters differently as they gradually gained experience evaluating the one hundred subjects’ CCTA, VIE, and ICA images, as reflected in the intra- and inter-observer variability. Furthermore, in our study, the stents’ sizes had a significant impact on the diagnostic accuracy for both CCTA and VIE, which was expected since smaller diameter stents are known to affect the diagnostic accuracy of CCTA [17]. Future studies, possibly utilizing images from dual-source CT [33], which has shown promising results in all size stents, may be warranted. Lastly, ICA was used as the reference method in our study, but VIE could be better validated with other intravascular imaging modalities, such as intravascular ultrasound (IVUS) [34] and optical coherence tomography (OCT) [35], which can provide better information on plaque conditions. Further studies should be conducted to assess the comparability of these intravascular imaging modalities.

## 5. Conclusions

Our study showed that VIE can be a helpful tool when evaluating coronary arteries with stents and plaques using CCTA, significantly improving the specificity, accuracy, and positive predictive value. This advantage may ultimately benefit patients by improving the diagnostic accuracy of CCTA and reducing unnecessary invasive procedures. Thus, CCTA-generated VIE could be incorporated into standard CCTA during the diagnostic assessment of coronary stents or plaques.

## Figures and Tables

**Table 1:** Diagnostic performance of CCTA versus VIE in detecting either in-stent restenosis or coronary artery disease in 902 vessel segments with stents or plaques, using ICA as the gold standard.

	CCTA	VIE	*p* Value
Sensitivity	77/82 = 93.9% (86.3–98.0%)	74/82 = 90.2% (81.7–95.7%)	0.563
Specificity	789/820 = 96.2% (94.7–97.4%)	805/820 = 98.2% (97.0–98.9%)	0.025
Accuracy	866/902 = 96.0% (94.5–97.2%)	879/902 = 97.7% (96.2–98.4%)	0.046
Positive predictive value	77/110 = 70.0% (60.5–78.4%)	74/89 = 83.1% (73.7–90.4%)	0.047
Negative predictive value	787/792 = 99.4% (98.5–99.8%)	805/813 = 99.0% (98.1–99.6%)	0.610
kappa value	0.7790.713–0.847	0.8510.791–0.911	0.112

**Table 2:** Diagnostic performance of CCTA versus VIE in detecting in-stent restenosis in 193 stented vessels, using ICA as the gold standard.

	CCTA	VIE	*p* Value
Sensitivity	22/23 = 95.7% (78.1–99.9%)	20/23 = 87.0% (66.4–97.2%)	0.608
Specificity	159/170 = 93.5% (88.7–96.7%)	166/170 = 97.6% (94.1–99.4%)	0.113
Accuracy	181/193 = 93.8% (89.4–96.8%)	186/193 = 96.9% (92.7–98.5%)	0.347
Positive predictive value	22/33 = 66.7% (48.2–82.0%)	20/24 = 83.3% (62.6–95.3%)	0.269
Negative predictive value	159/160 = 99.4% (96.6–99.9%)	166/169 = 98.2% (94.9–99.4%)	0.623
kappa value	0.7510.622–0.881	0.8300.709–0.941	0.391

**Table 3:** Diagnostic performance of CCTA versus VIE in detecting coronary artery disease in 115 vessel segments with calcified plaques, using ICA as the gold standard.

	CCTA	VIE	*p* Value
Sensitivity	3/4 = 75.0% (19.4–99.4%)	2/4 = 50.0% (6.8–93.2%)	0.999
Specificity	98/111 = 88.3% (80.8–93.6%)	106/111 = 95.5% (89.8–98.5%)	0.085
Accuracy	101/115 = 87.8% (80.4–93.2%)	108/115 = 93.9% (87.9–97.5%)	0.170
Positive predictive value	3/16 = 18.8% (4.1–45.7%)	2/7 = 28.6% (3.7–71.0%)	0.621
Negative predictive value	98/99 = 99.0% (94.5–99.9%)	106/108 = 98.1% (93.5–99.8%)	0.999
kappa value	0.2590.109–0.498	0.3340.094–0.573	0.743

**Table 4:** Diagnostic performance of CCTA versus VIE in detecting coronary artery disease in 85 vessel segments with mixed plaques, using ICA as the gold standard.

	CCTA	VIE	*p* Value
Sensitivity	31/34 = 91.2% (76.3–98.1%)	31/34 = 91.2% (76.3–98.1%)	0.999
Specificity	45/51 = 88.2% (76.1–95.6%)	47/51 = 92.2% (81.1–97.8%)	0.739
Accuracy	76/85 = 89.4% (80.9–95.0%)	78/85 = 91.8% (83.8–96.6%)	0.793
Positive predictive value	31/37 = 83.8% (68.0–93.8%)	31/35 = 88.6% (73.3–96.8%)	0.806
Negative predictive value	45/48 = 93.8% (82.8–98.7%)	47/50 = 94.0% (83.5–98.8%)	0.999
kappa value	0.7830.662–0.903	0.8290.708–0.949	0.617

**Table 5:** Diagnostic performance of CCTA versus VIE in detecting coronary artery disease in 31 vessel segments with non-calcified plaques, using ICA as the gold standard.

	CCTA	VIE	*p* Value
Sensitivity	21/21 = 100.0% (83.9–100.0%)	21/21 = 100.0% (83.9–100.0%)	0.999
Specificity	7/10 = 70.0% (34.8–93.3%)	8/10 = 80.0% (44.4–97.5%)	0.999
Accuracy	28/31 = 94.3% (74.3–98.0%)	29/31 = 93.6% (78.6–99.2%)	0.999
Positive predictive value	21/24 = 87.5% (67.6–97.3%)	21/23 = 91.3% (72.0–98.9%)	0.999
Negative predictive value	7/7 = 100.0% (59.0–100.0%)	8/8 = 100.0% (63.1–100.0%)	0.999
kappa value	0.7600.641–0.880	0.8440.710–0.966	0.612

**Table 6:** Diagnostic performance of CCTA versus VIE in detecting coronary artery disease in 424 vessel segments with stents, CP, MP, or SP, using ICA as the gold standard.

	CCTA	VIE	*p* Value
Sensitivity	77/82 = 93.9% (86.3–98.0%)	74/82 = 90.2% (81.7–95.7%)	0.563
Specificity	309/342 = 90.4% (86.7–93.3%)	327/342 = 95.6% (93.9–97.5%)	0.011
Accuracy	386/424 = 91.0% (87.9–93.6%)	401/424 = 94.6% (92.0–96.5%)	0.063
Positive predictive value	77/110 = 70.0% (60.5–78.4%)	74/89 = 83.1% (73.7–90.3%)	0.047
Negative predictive value	309/314 = 98.4% (96.3–99.5%)	327/335 = 97.6% (95.4–99.0%)	0.658
kappa value	0.7460.675–0.826	0.8320.764–0.901	0.092

## Data Availability

The data are not publicly available to maintain the privacy of study subjects.
